# Unveiling resistance mechanisms to EGFR inhibitors in cholangiocarcinoma

**DOI:** 10.18632/oncotarget.26403

**Published:** 2018-12-18

**Authors:** Javier Vaquero, Cindy Lobe, Laura Fouassier

**Affiliations:** Laura Fouassier: Sorbonne Université, INSERM, Saint-Antoine Research Center, Paris, France

**Keywords:** EGFR, cholangiocarcinoma

Cholangiocarcinoma (CCA) is a very aggressive cancer of the biliary tract. The only curative treatment currently offered to CCA patients is the surgical resection, which is limited to a minority of them (∼35 %) [1]. For patients diagnosed at late stages with inoperable CCA, the sole validated chemotherapy is based on gemcitabine and platinum salt regimen [2]. After this first-line of treatment, there are no other approved therapies for CCA treatment. Although knowledge on signaling pathways involved in CCA progression has significantly advanced allowing the discovery of actionable targets, there are still no approved targeted therapies for CCA treatment. Failure in using targeted therapies in CCA cure is partly explained by the high therapeutic resistance of biliary tumors, which molecular mechanisms remain fragmented [3].

Among signaling pathways, numerous studies including ours have highlighted a role of tyrosine kinase receptor EGFR (epidermal growth factor receptor or HER1) and its related members HER2, 3 and 4 in CCA progression [4]. Although preclinical studies have demonstrated a potential inhibitory effect of anti-EGFR drugs on CCA growth, none of these drugs, comprising either small molecules (erlotinib, gefitinib) or monoclonal antibodies (cetuximab, panitumimab), have shown a real benefit once tested in patients with advanced CCA, questioning the use of anti-EGFR in the treatment of CCA. Despite the disappointing results, drugs targeting EGFR are still being tested in clinical trials in patients with advanced biliary tract cancer without knowing the cause of anti-EGFR ineffectiveness.

Resistance mechanisms to anti-EGFR have been extensively studied in other cancers and several tracks have been proposed to explain the therapeutic failure of anti-EGFR. The most frequent mechanism of primary resistance is the presence of mutations in EGFR downstream pathway genes such as *KRAS*, which are common in CCA (22 to 42%) depending on tumor localization. Although not formally demonstrated, *KRAS* mutations do not appear to be involved in the lack of response to anti-EGFR [5]. In another study where only CCA patients without *KRAS* mutation were selected, anti-EGFR therapy did not add any benefit in survival to chemotherapy alone [6]. Further studies are now required and preclinical investigations using patient-derived xenograft (PDX) model of CCA bearing *KRAS* mutation could bring a valuable tool to deciphering resistance mechanisms to targeted therapies [7]. Additionally, mutations in *NRAS*, *BRAF*, *PI3KCA* and even in *EGFR* do not seem to play a role in the inefficiency of anti-EGFR treatment in CCA patients [8].

Activation of alternative signaling pathways in response to treatment is one of the mechanisms involved in resistance to targeted therapies, and in this case, it is named secondary (or acquired) resistance. No study had been conducted so far to understand the mechanisms underlying secondary resistance to anti-EGFR treatment in CCA. Our recent preclinical study helped to clarify this point [9]. In order to mimic *in vitro* long-term treatment of CCA with anti-EGFR, human CCA cell lines were treated with erlotinib, an EGFR tyrosine kinase inhibitor, for several weeks. During treatment, a drastic phenotypic change of the cells was observed, with the acquisition of epithelial-mesenchymal transition (EMT) and cancer stem cell properties. Interestingly, the phenotypic switch was confirmed in resistant cells irrespective of their mutational status in EGFR pathway; some had *KRAS* or *BRAF* and none had *EGFR* mutations in the tyrosine kinase domain. As EGFR pathway was well inhibited, an alternative signaling pathway was reported to be upregulated in resistant CCA cells, comprising insulin-like growth factor (IGF) 1 receptor (IGF1R), insulin receptor (IR), and IGF2, a ligand of IGF1R/IR. Then, a direct link was established between the activation of IGF2/IGF1R/IR pathway and EMT and stemness in resistant CCA cells. Using dual tyrosine kinase inhibitor (linsitinib) or siRNA strategy to block IGF1R/IR, we were able to demonstrate that these receptors were at the origin of the phenotype switch of erlotinib-resistant CCA cells.

To go further, *in vivo* investigations were undertaken in nude mice, a mice strain allowing engraftment of human cells and recapitulating the microenvironment of human tumors [10]. Surprisingly, tumors developed from resistant cells had a microenvironment enriched in α-SMA-positive cells, *i.e.* cancer-associated fibroblast (CAF), compared to parental cells, suggesting a potential education of stromal cells (originating from mice) by resistant tumor cells. This aspect of research would deserve to be investigated in a deeper way knowing the importance of microenvironment in tumor progression and therapeutic resistance. Cotreatment of the mice bearing resistant tumors with anti-EGFR and anti-IGF1/IR had not only a drastic inhibitory effect on tumor growth but also on stroma by decreasing its amount with less collagen deposition and α-SMA-positive cells.

One major feature of CCA is its dense desmoplastic stroma consisting predominantly of CAF. CAF can contribute in several ways to therapeutic resistance. In the case of anti-EGFR resistance in CCA, an expression of IGF2 in CAF was highlighted in erlotinib-resistant tumors. With the non-access of CCA samples from patients treated with anti-EGFR drug, the expression of IGF2 in human was checked and confirmed only in non-treated patients. By producing IGF2, CAF may target IGF1R/IR axis in a paracrine manner supporting resistance to anti-EGFR in CCA. But IGF2 may act also in an autocrine way on CAF because they express IGF1R. Indeed, IGF2 was able to stimulate hepatic myofibroblast proliferation, an effect inhibited by linsitinib [9].

In conclusion, the use of targeted therapies for the treatment of CCA patients remains challenging because of innate and acquired resistance mechanisms. Nevertheless, new generation of EGFR tyrosine kinase inhibitors are currently being tested to bypass this resistance. However, most recent studies have already suggested the emergence of new resistance mechanisms for these last generation of drugs. Therefore, new therapies and combinatory regimens must be developed to improve CCA treatment, a highly chemoresistant tumor. In order to success in this endeavor, it is mandatory to consider not only cancer cells but also stromal cells that have a fundamental role in CCA progression and drug resistance.
